# PROTOCOL: Effects of guaranteed basic income interventions on poverty‐related outcomes in high‐income countries: A systematic review

**DOI:** 10.1002/cl2.1281

**Published:** 2022-10-06

**Authors:** Anita Rizvi, Vivian Welch, Marcia Gibson, Patrick R. Labelle, Christina Pollard, George A. Wells, Elizabeth Kristjansson

**Affiliations:** ^1^ School of Psychology, Faculty of Social Sciences University of Ottawa Ottawa Canada; ^2^ Methods Centre Bruyère Research Institute Ottawa Canada; ^3^ MRC/CSO Social and Public Health Sciences Unit University of Glasgow Glasgow UK; ^4^ Library University of Ottawa Ottawa Canada; ^5^ School of Population Health, Faculty of Health Sciences Curtin University Bentley Australia; ^6^ School of Epidemiology and Public Health, Faculty of Medicine University of Ottawa Ottawa Canada

## Abstract

This is the protocol for a Campbell systematic review. The objectives are as follows: to appraise and synthesize the available quantitative evidence on GBI interventions in high‐income countries, for the purpose of comparing the relative effectiveness of specific forms of GBI for alleviating poverty.

## 1 BACKGROUND

### 1.1 The problem, condition, or issue

#### 1.1.1 Poverty in high‐income countries

Although the concept of poverty in high‐income countries seems like a contradiction in terms, there are nonetheless many people in these countries who rely on social assistance benefits, subsidized housing, donated clothing, and food banks to make ends meet. The incongruity of experiencing poverty in countries that are considered to be wealthy can be explained in part by the definition of a high‐income country: one that has a gross national income (GNI) per capita of US$12,696 or more (World Bank, 2022). Since the per capita amount is calculated by dividing the gross national income by the country's population, it doesn't provide any information on the distribution of the income within the population or indicate how many of its citizens are unable to afford a basic standard of living.

While it is expected that some people in the free‐market economies of high‐income countries will earn more money than others, income inequality has increased in most developed countries since 1990 (United Nations, 2020). Also, the proportion of the population in the middle‐income class (having 75%–200% of the national median household income) has declined since the mid‐1980s in most developed countries, while the size of the lower‐income class (below 75% of the national median household income) has grown in most (OECD, 2019). In contrast, due to strong economic growth in developing countries in the last two decades, the size of the global middle class has nearly doubled or tripled in that time, depending on the measure used (Versace, 2021). One factor in these divergent trends between higher‐income and lower‐income countries is the outsourcing of manufacturing by developed countries in recent decades, combined with technological advancement that has displaced routine‐based jobs, while increasing computing power and artificial intelligence is also placing non‐routine jobs at risk (OECD, 2021a).

According to the International Labour Organization (ILO), 22% of people in developed countries (more than 300 million) were considered poor in 2012, with an income of less than 60% of the national median—and since then, various indicators have shown poverty rates to be either unchanged or, in the case of the European Union (EU), trend higher after the 2008 global financial crisis (ILO, 2016). Based on the poverty threshold of the Organisation for Economic Co‐operation and Development (OECD), which is 50% of the national household median income, the poverty rates in developed countries have remained fairly stable between 2008 and 2019, ranging from 5.6% in Czechia (Czech Republic) to 18% in the United States (OECD, 2022). This data also shows the poverty rate for children (0–17 years old) in the United States and Spain to be the highest among developed countries, at 21%. (It should be noted that all the figures above refer to relative poverty, based on median incomes in these countries, and not to absolute poverty which is associated with problems such as malnutrition, unsafe drinking water, and lack of basic education; Peer, 2021).

Considering the basic material needs of food and shelter can also shed light on the prevalence of poverty, and these needs are unmet—either temporarily or chronically—for many people in high‐income countries. Because homelessness involves complex underlying factors besides not being able to afford housing, such as addictions, abusive relationships, and mental illness, this experience of poverty is outside the scope of this review, but has been addressed in others (e.g., Aubry, 2020; Nilsson, 2019). Inadequate access to food, on the other hand, is directly related to financial means in high‐income countries, as reflected in commonly used definitions of food insecurity: “a lack of available financial resources for food at the household level” (Hunger & Health, 2022), “[not] having physical and economic access to sufficient healthy food at all times” (Department for Environment, Food & Rural Affairs, 2021), and “the inadequate or insecure access to food because of financial constraints” (Tarasuk & Mitchell, 2020).

Over the past five decades, there has been a proliferation of food banks (“food pantries” in the United States) in all high‐income countries; however, because of their dependence on charitable donations, food banks are limited in their capacity to alleviate food insecurity (Loopstra, 2018). The prevalence of food banks in high‐income countries is an important factor in relation to poverty because the people who rely on food banks for assistance are typically in the most food‐insecure categories (moderately or severely food‐insecure) and have lower incomes than food‐insecure people who do not rely on food banks (Tarasuk, 2020).

#### 1.1.2 Policies and programs for reducing poverty

Social justice advocates have long asserted that poverty reduction is a moral obligation of the state which can be achieved by a fairer distribution of wealth (Barder, 2009; Standing, 2019). Although various types of support have been provided by the state to people in poverty since ancient times, the modern concept of social welfare emerged in the late 19th century in Germany under Chancellor von Bismarck, based on the precept that people facing poverty and distress should receive assistance from the state, not as a matter of charity but as a right (Rose, 1985). Other high‐income countries followed suit during the 20th century, implementing social assistance programs to alleviate poverty after the Great Depression (Trattner, 2007). In the United Kingdom during the Second World War, economist Sir William Beveridge wrote a report for the government which called for a “revolution” in the direction of Britain's welfare state and laid out a comprehensive set of social assistance programs, ranging from child benefits to pensions and funeral allowances. The Beveridge Report expanded on programs introduced by Lloyd George and Churchill three decades earlier and provided the blueprint for modern welfare in the United Kingdom (Day, 2017; Wheeler, 2015). Similarly, the Marsh Report of 1943 provided the foundation for the current social security system in Canada, by proposing measures similar to Beveridge's (a mentor of Marsh) and adding elements such as an employment program and health care insurance (Policy Options, 2004).

The cost of social assistance programs in high‐income countries is equivalent to between 12% and 31% of the gross domestic product (GDP), depending on the country (OECD, 2020). The generosity of social assistance also varies over time, with cutbacks being common during economic recessions due to politicians being pressured to support workers not “shirkers” (Romano, 2015).

Social welfare programs were found to reduce poverty significantly in high‐income countries between 1960 and 1991 (Kenworthy, 1999). Since then, however, welfare reforms—often called “workfare” because of their emphasis on transitioning social assistance recipients into the workforce—have been blamed by critics for reversing the poverty reduction trend by cutting benefits to the unemployed, including single mothers, and requiring them to accept precarious, low‐paying jobs (Carey & Bell, 2020; Widerquist et al., 2013). The increased conditionality of workfare may also result in additional stigma and shame for recipients who either remain unemployed, or those who are skilled and placed in menial, low‐paying jobs (Carey & Bell, 2020; Widerquist, 2013). Sanctions in the form of benefit cuts and interruptions are intended to increase compliance with the conditions of workfare programs (e.g., actively seeking work), but some studies have suggested that these sanctions can have detrimental effects on mental and physical health, debt, material hardship, and financial stress (Pattaro, 2022).

Because social assistance programs rely on a minimum income threshold to determine eligibility, transitioning to a low‐paying job with an income slightly above the threshold results in losing the benefit. Additionally, it may also mean losing in‐kind benefits such as a rent subsidy and dental care, so a person's net income may end up being even lower than the amount provided by social assistance (Wolfson, 2018).

A distinguishing feature of social assistance in many high‐income countries is the availability of various programs, offered by different levels of government and targeted at specific groups (e.g., people with disabilities, women with infant children) and specific needs (e.g., money for food or rent). This approach has been criticized as being a patchwork of programs that are confusing in terms of understanding eligibility criteria, and which fail to provide some categories of people with a subsistence‐level income (Koebel & Pohler, 2019; Wolfson, 2018). The complexity of the programs and uncertainty regarding eligibility also translates into high levels of non‐take‐up, which results in many people missing out on benefits that they are eligible to receive. Although non‐take‐up results in short‐term savings for the government, it may result in more costly downstream effects if it prevents people from affording early medical treatment or paying for a better education for their children (Van Mechelen & Janssens, 2017).

The United Kingdom introduced a welfare reform called Universal Credit (UC) in 2012, which consolidates six previously separate programs (Winchester, 2021). To be eligible for UC, most recipients who are unemployed (except those with infant children) have to seek work or take training courses, and noncompliance such as missing an appointment with a work coach can lead to sanctions (UK Government, 2014). Some studies also suggest that the reforms of UC have led to an increase in poverty for single mothers (Carey & Bell, 2020).

One type of supplementary social assistance offered in many high‐income countries is in the form of refundable (or payable) tax credits, which provide cash benefits to eligible people with low incomes who file income tax returns. However, this form of income supplement has been criticized as being insufficient, especially for people with low incomes and without children (Koebel & Pohler, 2019). In the United Kingdom, only two types of refundable tax credits are currently offered: a working tax credit and a child tax credit (UK Government, 2022), so unemployed people without dependent children are not eligible for either. Also, refundable/payable tax credits only reach those who file income tax returns, and the rate of non‐filing is as high as 20% among people with very low incomes (Robson & Schwartz, 2020).

#### 1.1.3 Universal basic income (UBI)

UBI has been proposed as a way to alleviate poverty (Hasdell, 2020) and to replace the current assortments of social assistance programs in high‐income countries, administered by different levels of government, which have been described as bureaucratic, costly, and stigmatizing (Koebel & Pohler, 2019; Reed & Lansley, 2016). UBI is “an income paid by a political community to all its members on an individual basis, without means test or work requirement” (Van Parijs, 2004, p. 8). More recently, additional dimensions of UBI have been specified: it is paid at regular intervals and as cash payments which recipients can spend in any way they choose (BIEN, 2020). The amount of the UBI payment should also be stable and predictable (Standing, 2021).

Proponents of UBI have criticized the reformed welfare programs of the past three decades as being fiscally unsustainable, overly intrusive, and inhibiting the agency of benefit recipients (Orrell, 2021). In terms of public opinion, a study in the United Kingdom and the United States found that the two main reasons cited in support of UBI were simplicity and efficiency of administration, and reducing stress and anxiety (Nettle et al., 2021).

Other important implications for UBI pertain to inequalities across socioeconomic status, race, ethnicity, and gender. Stressors such as financial difficulties, caring for disabled children or parents, and abusive relationships at work or home have damaging effects on mental and physical health, and these effects disproportionally impact women, racial/ethnic minorities, and people with low incomes (Thoits, 2010).

For women, UBI paid on an individual basis could potentially improve several areas of concern. Firstly, UBI would provide an income for women who perform work outside the formal labor market, such as caring for children and doing volunteer work, as well as for those who have personal care jobs which usually do not pay well. An individual‐level UBI would also reduce the financial dependency of spouses in abusive households, who currently are not eligible for social assistance if their spouse earns an income above the eligibility threshold (Bidadanure et al., 2018).

Poverty rates in high‐income countries are disproportionally high for black and Indigenous people as well as for other racial and ethnic minorities, often resulting from involuntary unemployment due to discrimination and lack of opportunities. UBI has been proposed since the 1960s by Martin Luther King, Jr., the Black Panther Party, and other advocates as a way to alleviate poverty due to systemic racism. Although UBI could potentially reduce income inequality along racial lines, there has not been much recent policy discussion on this topic (Bidadanure, 2019).

UBI is, however, receiving renewed attention due to rising income inequality and the changing nature of work due to automation and reductions in the quantity and quality of jobs (Gentilini, 2020; Hasdell, 2020). More recently, the economic disruptions brought about by the COVID‐19 pandemic have further prompted policy discussions on full‐scale UBI programs. On the other hand, the concept of UBI is also controversial and has been criticized for disincentivizing work and for being extremely costly, to the point that it could result in cuts to healthcare and education (Centre for Social Justice, 2018; Hoynes & Rothstein, 2019).

#### 1.1.4 Measuring poverty

Regardless of the type of poverty reduction approach that is implemented, a major challenge is evaluating the effectiveness of the approach. This is because a standardized method does not exist for measuring poverty—indeed, there has been considerable debate over which poverty indicators are most accurate and reliable (Cutillo, 2020; Meyer & Sullivan, 2012). Official poverty measures have traditionally been based on income, setting some minimum threshold as the poverty line, while newer poverty measures factor in the cost of living, or at least the cost of basic needs (Cutillo, 2020; Guio, 2016; Meyer & Sullivan, 2012). Simple income‐based measures are still commonly used and have been criticized as being outdated and that they measure income inequality, not poverty (Gupta & Theoharis, 2020; Konle‐Seidl, 2021). The Organisation for Economic Co‐operation and Development, for example, defines the poverty line as “half the median household income of the total population” in each country (OECD, 2021b). Because of the arbitrary poverty threshold of such measures, millions of people slightly above the poverty line live precariously—“just a $400 emergency away from poverty” (Gupta & Theoharis, 2020).

Consumption‐based measures, which use surveys to assess what goods and services individuals or households consume, have been proposed as a more accurate indicator of poverty. A comparison of various poverty measures in Europe found that consumption‐based poverty measures identified different groups as being poor, compared to income‐based measures, and that income had a low correlation with severe material deprivation (Cutillo, 2020). Similarly, a comparison of poverty measures in the United States, including the official poverty measure (OPM), found that a consumption‐based measure was more accurate in identifying people who were facing financial hardship—that is, low consumption was a better indicator than low income (Meyer & Sullivan, 2012). Consumption‐based measures can also identify those with incomes above the official poverty line, but who spend a large amount on health‐related expenses, which may cause difficulty in affording food and rent (Sarabia, 2016).

The inaccuracy of income‐based poverty measures, even when the cost of living is factored in, can be demonstrated by non‐monetary indicators of poverty. For example, in Canada the new‐for‐2016 official poverty measure, the Market Basket Measure (MBM), indicates that the percentage of Canadians living below the poverty line decreased considerably, from 15.0% in 2012% to 10.1% in 2019. Over almost the same period, however, the prevalence of food insecurity increased slightly, from 8.3% of households in 2011–2012 to 8.7% in 2017–2018 (Statistics Canada, 2021). As well, the number of people aged 65 and older who visited food banks because they did not have enough money for food increased by 29.8% between 2016 and 2019 (Food Banks Canada, 2019). Official poverty measures also may not capture the impacts of food poverty on children, for whom food insecurity is not only associated with hunger and inadequate nutrition, but also with social, developmental, and health impacts that may persist into adulthood (Ramsey, 2011; Thomas, 2019).

Food insecurity has been proposed as a more accurate and sensitive indicator of poverty than measures based on income and estimates of the cost of living (Loopstra & Tarasuk, 2013; Power, 2016). Loopstra and Tarasuk observed a linear relationship between the severity of food insecurity and the odds of experiencing hardships such as not being able to pay rent and bills on time.

To examine the relationships of various types of material deprivation, Toppenberg (Toppenberg, 2017) constructed regression models using data from the US Census Bureau's 2015 Current Population Survey Food Security Supplement, and found that compromised health, education, standard of living, and housing were all better predictors of food insecurity than low income.

Recently, there has been increasing attention in the social sciences and policy research on the multi‐dimensional nature of poverty, which includes income poverty and material deprivation, as well as the psychological dimension of subjective financial stress (Schenck‐Fontaine & Panico, 2019). Other less tangible aspects of poverty, which income and consumption measures are not able to capture, are deficits in the areas of “voice, human security, isolation, dignity, lack of time, and subjective wellbeing” (Poverty Analysis Discussion Group, 2012; p. 5).

Interestingly, multidimensional poverty indices have been adopted in many developing countries as official poverty measures, incorporating the dimensions mentioned above, as well as: basic services, environment, personal safety from violence, and social inclusion (ITWG 2021). Non‐governmental bodies such as the United Nations Development Programme (UNDP) and the International Fund for Agricultural Development (IFAD) have also developed multidimensional poverty measures, as has the United Nations Children's Fund (UNICEF) to assess poverty of children (SDSN, 2019).

The European Union (EU) adopted a new official poverty measure in 2010 which is described as multidimensional (SDSN, 2019; Whelan, 2014); however, it only includes three indicators: relative income (60% of the national median), employment, and material deprivation.

In this review, we will examine basic income interventions for reducing poverty, assessed using traditional income‐based poverty measures as well as alternative and novel measures—based on food insecurity, consumption, material deprivation, subjective financial stress, and other physical, social, and psychological dimensions of poverty that are reported in studies—to assess and compare the effectiveness of different variants of a guaranteed basic income.

### 1.2 The intervention

A truly universal basic income policy has never been implemented in high‐income countries (Gentilini, 2020; Gibson, 2020). Thus, our review will examine basic income interventions which include some features of UBI, as described below. These quasi‐UBI approaches are known by various terms such as: basic income guarantee (BIG), guaranteed annual income (GAI), unconditional cash transfer (UCT), and negative income tax (NIT). All of these variations share the common attribute of monetary benefits that would be guaranteed by the state (Van Parijs & Vanderborght, 2017), so we will use the term “guaranteed basic income” (GBI) in this review to cover all types of basic income interventions. The shorter term “basic income” is often used in the literature as a short form of “universal basic income”; therefore, we will use the term “guaranteed basic income” (GBI) to avoid confusion. For the meaning of basic, we will use the two interpretations outlined by Hoynes and Rothstein (Hoynes & Rothstein, 2019): (1) an amount sufficient to pay for one's basic needs, or (2) an amount given to each recipient that provides a base which can be supplemented by other forms of income.

We will also define the ‘regular’ and ‘predictable’ payment criteria of GBI as being paid at least once per year and in the same amount each time. Although these are not always considered core criteria of a basic income, we consider predictable, regular payments of a fixed amount to be essential if GBI is used as an intervention to reduce poverty. Not knowing if the next payment will cover the same expenses as the previous one may cause anxiety and apprehension for the recipient, which could aggravate the experience of poverty. Because some programs, described as a type of basic income, are based on dividends which can change in amount over time (e.g., from oil or casino revenues), we will include only those studies in which the amount received varies by less than 10% during the study period (i.e., the lowest amount received by each recipient must be at least 90% of the highest amount received).

One form of GBI is a negative income tax (NIT), whereby people whose income is below their tax liability threshold would receive an amount from the government based on a prescribed tax rate. For example, if a person's employment income was $20,000 per year and they would have to pay tax on income over $30,000, then the $10,000 difference would be subject to a “negative tax” such that the government would pay some amount of money to this person. If the tax rate was 50%, this person would receive $5000 per year as the NIT benefit, resulting in a total income of $25,000. If on the other hand, the person had no income at all, the NIT benefit would be $15,000, so the person's total income would never fall below this amount and additional income would be subject to the NIT tax rate. In contrast, welfare benefits are cut dollar for dollar if the recipient earns more income, so there is less incentive for recipients to seek low‐paying jobs.

Some other forms of GBI also have a “take‐back” condition in the intervention whereby the benefit is reduced at a known, prescribed rate when there is additional income from employment or other sources; however, the benefit must include a minimum guaranteed amount that is paid unconditionally (i.e., not affected by changes in income or employment status). This guaranteed amount will serve to differentiate studies of GBI included in this review from those of existing social assistance programs, including those with “soft” (minimal) eligibility criteria.

In summary, we will include interventions that meet the following criteria: (1) regular payment intervals, (2) paid in cash (not in‐kind), (3) a guaranteed minimum amount received unconditionally, and (4) fixed (within 10%) or predictable amounts.

#### 1.2.1 A note on means testing

In this review, we distinguish between means testing that is used to determine eligibility for social assistance programs, versus means testing that is used to recruit participants for a GBI program, pilot, or experiment. For social assistance, means testing is conducted on an ongoing basis, to monitor eligibility and to adjust the amount of the benefit if required (e.g., reducing the benefit amount if employment income increases). We will include studies of GBI interventions if participants are enrolled based on low income, unemployment, or other means‐related factors, but not if the amount of the benefit is adjusted periodically based on those factors, with a dollar‐for‐dollar withdrawal rate in the benefit amount, as this would be similar to how conventional social assistance programs are administered. Similarly, we will exclude studies of interventions that involve ongoing means testing to reassess eligibility based on changes in the participants’ financial circumstances.

### 1.3 How the intervention might work

Proponents of GBI suggest that it is a preferable way to relieve poverty than conventional welfare programs for several reasons:

1. GBI would avoid the stigmatization inherent in conditional, means‐tested programs by offering the benefit to everyone within a community or at least everyone below a certain income threshold (Gentilini et al., 2020; Jenkins, 2019).
2. The means testing of applicants and scrutiny of recipients in welfare programs is labor‐intensive to conduct; these procedures are not necessary with GBI. Thus, it would be a more efficient method of poverty reduction (Widerquist et al., 2013; Yang et al., 2021).
3. GBI is a matter of social justice which addresses growing income inequality and fosters a fairer sharing of the public wealth accumulated over successive generations (Gentilini et al., 2020; Standing, 2021).

One drawback of welfare programs is that not everyone who is eligible ends up receiving the benefit. Many people do not apply for assistance because of the stigma and shame associated with welfare, while others may not realize they are eligible because of the complex requirements and procedures for enrollment (Bidadanure, 2019; Gentilini et al., 2020). Alternatively, because government programs are often targeted toward specific populations (e.g., families with children), some people do not qualify for assistance (Koebel & Pohler, 2019). Because everyone in the community would be eligible for GBI, or those people under some income threshold, these problems would be avoided, as everyone with a low income would be able to receive the benefit.

As noted above, analyses of poverty measures based on income have found that they may not be accurate indicators of poverty. Part of the reason for this could be that these measures are based on aggregated data and do not consider individual circumstances ‐ for example, people who are unemployed but living in affluent households would be grouped in the extremely poor category, based on their income. On the other hand, some people may have incurred large debts in the past which still cause financial hardship, but they wouldn't be counted as poor if they had incomes above the official poverty line. As pointed out by Meyer and Sullivan (Meyer & Sullivan, 2012, p. 116), “income‐based measures […] will not capture differences over time or across households in wealth accumulation, ownership of durable goods such as houses and cars, or access to credit.” As such, this review will examine studies of GBI interventions that use alternative measures, as described above, to assess their effectiveness for poverty reduction.

Food security was one outcome in a study of the Ontario Basic Income Pilot (OBIP) in Canada in 2018–2019, which provided a payment to recipients equal to 75% of the official poverty line, more generous than existing social assistance amounts. Over two thirds of the respondents in the study reported that their diet had improved, they skipped meals less often, ate more nutritious food, and accessed food banks less often (McDowell & Ferdosi, 2021).

The B‐MINCOME project in Barcelona targeted 1000 low‐income households (plus 383 households in the control group) from 2017 to 2019. This project involved various intervention arms, some of which meet our GBI criteria for inclusion in this review. All households received a guaranteed minimum income to cover basic living expenses, and 531 were also involved in one of four “active policies”: training and employment, social entrepreneurship, housing subsidies, and community participation. There were also four modalities of participation: (a) compulsory participation in one of the active policies, (b) participation not compulsory, (c) benefit amount is reduced if there is other, additional income, and (d) benefit amount not reduced with additional income. The study found statistically significant reductions in food insecurity for all the intervention types, particularly when the benefit was conditional on participation in an active policy or when the benefit wasn't reduced if there was additional income. There were also statistically significant reductions in material deprivation (a consumption‐based measure) for all intervention types except when participation in an active policy was compulsory (Laín, 2019).

### 1.4 Why it is important to do this review

We found the following reviews that included GBI‐like interventions in high‐income countries, including one review of other reviews:

- Hasdell (Hasdell, 2020) conducted a synthesis of reviews, published between 2011 and 2020, of interventions globally that included at least two features of UBI. Three reviews for low‐ and middle‐income countries were included that reported on food insecurity or material deprivation.
- Gentilini et al. (Gentilini, 2020) produced a guide published by the World Bank that examined interventions similar to UBI globally and included one study in Sub‐Saharan Africa that reported on food security. Effects on poverty were assessed using two measures which are based on income alone: the poverty headcount and the squared poverty gap.
- Gibson et al. (Gibson, 2020) conducted a scoping review of interventions similar to basic income in upper‐middle‐income and high‐income countries, which examined health outcomes. One qualitative study was included that reported increased food security.
- Günther (Günther, 2020) reviewed 60 articles as part of a Master's thesis on basic income schemes and experiments globally to evaluate income and employment elasticities.
- Gupta et al. (Gupta, 2021) conducted a review of basic income experiments globally and examined the effect of mitigating income poverty on mental health.
- Pinto et al. (Pinto, 2021) conducted a systematic review that identified 86 articles on 10 basic income interventions implemented globally to examine the various methods used to evaluate the effectiveness of the interventions.
- Yang et al. (Yang, 2021) reviewed 152 pieces of literature on basic income theories and empirical cases (15 studies globally) to analyze the relationship between conceptual definitions of basic income and how interventions have been implemented.

The current review will be the first to quantitatively evaluate the effectiveness of various forms of GBI for reducing poverty in high‐income countries, using food security level, consumption, material deprivation and multi‐dimensional poverty indicators as primary outcomes. Although other reviews have included outcomes related to various dimensions of poverty, this review will synthesize findings related to all relevant material, social, and psychological outcomes according to current multi‐dimensional conceptualizations of poverty.

#### 1.4.1 Policy relevance

Although guaranteed basic income as it is thought of today was first proposed by Thomas Paine in the 18th century, there has been a resurgence of support for GBI in recent decades by advocates in various fields: philosophy, economics, social policy, high‐tech, and notably, from opposing points on the political spectrum (Alston, 2017). However, a major obstacle to constructive policy debates on GBI is that the theoretical conceptualizations of basic income—usually the universal variety—do not quite align with the ways in which GBI programs, pilots, and experiments have been implemented in practice (Gentilini, 2020; Yang, 2021). The disassociation between theoretical conceptualizations and the actual designs of empirical GBI interventions, as well as the heterogeneity of these designs, makes it difficult to agree on principles to guide the development of full‐scale GBI programs (Gentilini, 2020; Yang, 2021). Because empirical GBI interventions only include some features of a true UBI and often enroll participants based on having income below some threshold, there is also ambiguity between the definitions of these interventions and those of liberal welfare programs. As well, the roles of various stakeholders—researchers, politicians, communities, news media—give rise to competing expectations which may result in misperceptions of the findings of GBI studies (Merrill, 2022). For these reasons, this review will attempt to develop a framework or rubric to facilitate the evaluation and comparison of various types of GBI interventions, so that empirical evidence can be more objectively assessed and synthesized and be more useful for policy discussions.

The inclusion of alternative and novel poverty measures in this review will also be relevant to public and social policy, particularly with respect to health and healthcare. The association between poverty and poor physical and mental health has been well documented (Boozary & Shojania, 2018; Gundersen & Ziliak, 2015; McLeod & Veall, 2006; Seligman & Schillinger, 2010). Income, however, was found to be a weak determinant of health in a large study by the United States Department of Agriculture, which reported that income was associated with 3 of 10 chronic diseases, while food insecurity was associated with all 10 (Gregory Christian & Coleman‐Jensen, 2017). Thus, if policymakers rely on official poverty measures based on income, with the assumption that poverty is being measured accurately, vulnerable populations may be overlooked if they are not identified by the poverty measure (Cutillo, 2020).

A review of GBI interventions is naturally relevant to discourses of public and social policy since the main goals of GBI are to reduce poverty and societal inequity. Moreover, GBI may benefit a specific population which does not qualify for regular welfare benefits: the “working poor” (Caputo, 2007; Koebel & Pohler, 2019; Riches & Tarasuk, 2014). While welfare eligibility has become more restrictive in recent decades, real income from employment has remained stagnant. According to the Economic Policy Institute (EPI, 2021), productivity and workers’ wages increased at almost the same rate in the United States from the 1940s until the early 1980s. Since then, while productivity has continued to grow at the same pace, increasing by 62% between 1980 and 2020, wages have only increased by 17.5% in these four decades. Over the same time, the income gap between the rich and the poor has grown much wider: household income for the lowest quintile, adjusted for inflation, remained essentially unchanged between 1973 and 2015, whereas for the wealthiest 5% it increased by 60% (Stone et al., 2020). This suggests that most of the wealth generated by the increased productivity during recent decades has gone to the rich. The reasons for this include labor laws that favor corporations over unions, decreasing tax rates for the wealthy, and small increases in the minimum wage which have not kept pace with inflation (EPI, 2021). These factors, combined with workfare programs placing more people into low‐paying jobs, have resulted in increasing numbers of the “working poor”.

## 2 OBJECTIVES

This systematic review will aim to appraise and synthesize the available quantitative evidence on GBI interventions in high‐income countries, for the purpose of comparing the relative effectiveness of specific forms of GBI for alleviating poverty. As such, we will seek to answer the following research questions:

- What are the effects of various forms of a guaranteed basic income (GBI) on poverty and food security in high‐income countries?
- Is there sufficient evidence available to determine a minimum amount of GBI to effect significant reductions in poverty?
- Does GBI affect subgroups within the population differently (by age, ability, education, gender, ethnicity, etc.)?
- How do estimated effect sizes vary with the type of poverty measure used (income based, consumption based, multi‐dimensional measures, and food security level)?
- What is the relationship between the various measures of poverty (i.e., which ones predict similar effects across different types of interventions)?

## 3 METHODS

We will conduct and report this review according to the Methodological Expectations of Campbell Collaboration Intervention Reviews (MECCIR) guidelines (Methods Group, 2019, 2019). Due to the relevance of the review topic to societal equity, we will also follow the PRISMA‐Equity reporting guideline (Welch et al., 2012). As well, we will consult the AMSTAR 2 critical appraisal instrument (Shea et al., 2017), intended to assist policymakers in assessing the quality of systematic reviews, to ensure that this review clearly addresses all the relevant criteria.

### 3.1 Criteria for considering studies for this review

#### 3.1.1 Types of studies

The review will include primary studies that collect and analyze quantitative data on poverty‐related effects of GBI interventions. We will exclude qualitative studies (e.g., case reports, narrative reports of interviews or focus groups) as well as any literature that refers to primary research reports or findings, such as reviews and compilations of studies, books, news and magazine articles, editorials, opinion pieces, or blogs.

We will include quantitative studies with any of the following designs:

- Randomized controlled trial (RCT)
- Cluster randomized controlled trial (cRCT)
- Controlled before and after (CBA)
- Regression discontinuity design (RDD)
- Interrupted time series (ITS) with at least three time points before, three time points after, and a time‐series analysis
- Cohort (prospective or retrospective, including cross‐sectional) with or without a control group, and with at least two repeated outcome measures

Cross‐sectional studies using data from a single time point will be excluded as they do not examine change over time in a particular cohort.

We will include all longitudinal quasi‐experimental designs even if they lack statistical controls; however, the more rigorous designs will likely be deemed to have higher internal validity, based on the risk‐of‐bias assessments.

#### 3.1.2 Types of participants

We will include studies that involve any group of people in developed high‐income countries (defined under “Types of settings” below). Children will be included since some studies examine outcomes for the children of parents or guardians who receive GBI benefits.

#### 3.1.3 Types of interventions

We will include any cash transfer programs for adults (18+ years old) in high‐income countries that meet our four criteria for GBI interventions: (1) regular payment intervals, (2) paid in cash (not in‐kind), (3) a guaranteed minimum amount received unconditionally, and (4) fixed (within 10%) or predictable amounts.

Refundable/payable tax credits will be excluded because they are either small in amount (i.e., not enough to provide an income “base”) or they are conditional (e.g., being employed, enrolled in a training program, having children of a certain age, caring for adults, or having a disability).

GBI benefits can be paid on an individual or household basis. The interventions can be administered by governments (usually as pilot projects) or by non‐governmental or civil society organizations for research purposes. In studies that include control groups, usual care would be in the form of conventional government assistance programs for participants who are eligible to receive them or no government assistance for those who aren't.

#### 3.1.4 Types of outcome measures

Below are descriptions of the outcomes of interest for this review. We will not exclude studies on the basis of outcome measures, as some studies may report other poverty‐related outcomes that are important to include in this review.

The primary outcome of food security level is typically assessed using survey‐based, self‐reported, and validated measures of food security that include questions on various aspects of food security. The survey responses are quantified using scoring rubrics which vary among high‐income countries. Examples of measures include the UN FAO Food Insecurity Experience Scale (FIES), used in Europe and the United Kingdom, and the Household Food Security Survey Module (HFSSM) in the United States and Canada (same survey but with differing food insecurity classification thresholds).

For measuring poverty, we will include official national measures such as the United States’ Official Poverty Measure (OPM) and Supplemental Poverty Measure (SPM), Canada's Market Basket Measure (MBM), OECD relative poverty measure, the poverty gap index, as well as consumption‐based indicators such as the Household Budget Survey (HBS), Consumer Expenditure (CE) Survey, and measures of deprivation such as the European Union's Material Deprivation (MD) Index, and other measures of ability to cover basic needs.

For the secondary outcomes, all measures below will be included. Some outcomes such as weight and height measures, used to determine body mass index (BMI), will be measured using instruments or self‐reporting, while other outcomes such as self‐reported health status will be measured using validated scales (e.g., the SF‐12 Survey for physical and mental health). Some secondary outcomes may be individual components of poverty indicators (e.g., food expenditure would be a component of a consumption measure).

##### Primary outcomes

- Food security level (using survey‐based, validated measures, as described above)
- Poverty level assessed using instruments intended or designed to measure poverty: income‐based official poverty measures, and alternative/novel measures based on material hardship/deprivation, the consumption level of goods and services, as well as multi‐dimensional measures of physical, social and/or psychological wellbeing

##### Secondary outcomes

- Food expenditure
- Self‐reported physical and mental health
- Body mass index (BMI)
- BMI for age
- Mid‐upper arm circumference (MUAC)
- Birth weight of children
- Cognitive development, literacy, and numeracy of children
- School/training program enrollment (children and adults)
- Employment/self‐employment status/labor force participation
- Individual/household earnings

#### 3.1.5 Duration of follow‐up

No restrictions will be placed on the duration of follow‐ups.

#### 3.1.6 Types of settings

We will include studies from any setting in developed high‐income countries, according to the classification of the United Nations Department of Economic and Social Affairs (UN DESA, 2022). Some countries that fall under the high‐income country category of the World Bank (e.g., Chile, Oman, Saudi Arabia) are classified by the International Monetary Fund (IMF, 2022) and UN DESA as emerging market economies, developing economies, and/or developing countries. Because these terms are commonly used to refer to low‐ or middle‐income countries in research articles, reports, and policy discussions, we will only include studies from high‐income countries that are classified by UN DESA as developed countries, to avoid potential confusion.

### 3.2 Search methods for identification of studies

This review will focus on studies that investigate GBI programs and initiatives implemented in developed high‐income countries. The search strategy proposed for this review builds on those used in previous reviews on GBI (Gibson, 2020; Pinto, 2021). Searches using both keywords and database‐specific controlled vocabulary will be conducted in relevant databases, and complementary searches will be done to identify additional studies as well as pertinent gray literature.

#### 3.2.1 Electronic searches

Searches will be conducted in subject‐specific and multidisciplinary databases to identify relevant published studies to include in this review. Searches will be executed by PRL in the following databases (in alphabetical order): APA PsycInfo (Ovid), Academic Search Complete (EBSCOhost), Business Source Complete (EBSCOhost), Cochrane CENTRAL (Ovid), CINAHL (EBSCOhost), EconLit (EBSCOhost), Embase (Ovid), Global Health (EBSCOhost), International Bibliography of the Social Sciences (ProQuest), International Political Science Abstracts (EBSCOhost), MEDLINE (Ovid), PAIS Index (ProQuest), ProQuest Dissertations & Theses Global (ProQuest), Sociological Abstracts (including Social Services Abstracts, ProQuest), Web of Science (all indexes), Worldwide Political Science Abstracts (ProQuest).

Database limits will not be used, and no restrictions related to languages, dates, or publication types will be imposed when searching the above resources.

An initial, sensitive search strategy was developed for MEDLINE (Ovid). Given the scope of this review, the research librarian and principal investigator determined that searching broadly for studies related to GBI would suffice and that no additional concepts would be included and combined. To assess its effectiveness, the strategy was peer‐reviewed by another research librarian following the Peer Review of Electronic Search Strategies (PRESS) guideline for systematic reviews (McGowan, 2016). This search strategy will then be translated for the other databases using pertinent subject headings, where applicable, as well as appropriate search syntax. The MEDLINE strategy is available in Supporting Information: Appendix 1.

In addition to using the above resources, searches will be done in the Cochrane Database of Systematic Reviews (Ovid), the Campbell Systematic Reviews journal (Wiley), the Social Systems Evidence database (McMaster University), and Epistemonikos (via the Cochrane Library) to identify relevant review articles.

Included studies will be added to Zotero, which integrates notifications from Retraction Watch, to determine if any of them have been retracted. Each included study will also be accessed on its original publisher platform to verify whether any corrections or updates were made since the original text was published.

#### 3.2.2 Searching other resources

Various approaches will be used to identify relevant gray literature.

To find conference proceedings, both the Science and the Social Sciences & Humanities editions of the Conference Proceedings Citation Index will be searched (at the same time as the other indexes in the Web of Science). In addition, reviewers will consult specific conference websites such as the BIEN Congress (https://basicincome.org/congress-papers/) and the Annual Basic Income Guarantee (BIG) Conference (https://usbig.net/2022congress/) to browse proceedings and presentations from the last 5 years.

Relevant graduate research will be found through searches done in ProQuest Theses & Dissertations Global (ProQuest) as well as in many of the databases identified above which also index theses and dissertations.

Government information and other types of gray literature such as white papers and preprints are more challenging to find, but the websites and catalogues of the following organizations will be targeted: United Nations (via ODS; https://documents.un.org/prod/ods.nsf/home.xsp, and via the UN Digital Library; https://digitallibrary.un.org/), World Bank (via the Open Knowledge Repository; https://openknowledge.worldbank.org/, and via its eLibrary; https://elibrary.worldbank.org/), World Health Organization (https://www.who.int/publications), Social Science Research Network (https://www.ssrn.com/), National Bureau of Economic Research (https://www.nber.org/), Research Papers in Economics (http://repec.org/), Institute of Labor Economics (https://www.iza.org/), and OECD (via its iLibrary (subscription access)). Targeted, specific searches of government websites of Group of seven high‐income countries will also be conducted (Canada; https://publications.gc.ca/site/eng/home.html, France; https://www.gouvernement.fr/, Germany; https://www.bundesregierung.de/breg-en/service/information-material-issued-by-the-federal-government, Italy; no search option for English, Japan; https://www.japan.go.jp/publications/index.html, the United Kingdom; https://www.gov.uk/official-documents, and the United States; https://www.govinfo.gov/).

In addition to searching for gray literature, other means of identifying studies will be used and are described below.

Reference lists from relevant knowledge syntheses (systematic and non‐systematic reviews) as well as those from included primary studies will be examined to see if other studies should be considered. Citation searching of included articles will also be conducted using Google Scholar (https://scholar.google.com/).

Once title and abstract screening is complete, journal titles of references eligible for full‐text review will be analyzed to select the five journals that appear most frequently. These journals will then be hand searched by looking specifically at each one's table of contents for the past 5 years.

The corresponding authors of included studies will be contacted by email and will be provided with a list of included articles along with the inclusion criteria for the review. They will be asked if they are familiar with any additional studies that might be relevant. In addition, authors of conference presentations will be contacted to see if their research has been published as articles or if they have data or results that they are willing to share or that will be published in the near future.

### 3.3 Data collection and analysis

#### 3.3.1 Description of methods used in primary research

GBI interventions (programs, experiments, and pilot studies) have typically been carried out within selected geographic regions with participants whose income falls below a certain threshold amount. Some studies employ a saturation approach where every eligible person in the community who enrolls receives the benefit, so that community‐level effects can be examined. Although the types of outcomes are numerous, data are usually collected using surveys, and sometimes analyzed by incorporating administrative data.

Some basic income experiments are conducted as randomized controlled trials (RCTs) with intervention and control groups, while others are of a quasi‐experimental (observational) nature, some using statistical controls such as propensity score matching to reduce bias.

#### 3.3.2 Selection of studies

All stages of screening references will be conducted with the use of Covidence, an online tool designed to streamline certain stages of review projects (https://www.covidence.org/). A summary of the inclusion and exclusion criteria (see Supporting Information: Appendix 2) will be posted on Covidence for reference. The selection of studies will begin with title and abstract screening, performed independently whereby each reference will be seen by two reviewers. In case of disagreement, the decision on including the reference will be made by the principal investigator. The same process will be used at the full‐text screening stage to determine the eligibility of the references which are retained after title and abstract screening. The reasons for excluding references at this stage will be recorded and presented in a PRISMA flow diagram.

Both screening phases will be subject to a pilot to ensure that the inclusion and exclusion criteria are applied consistently by all reviewers. Twenty‐five randomly selected references will be used for the title and abstract pilot, and ten randomly selected references will be used for the full‐text pilot. Reviewers will be able to provide feedback in the pilot forms at both stages regarding the clarity of the inclusion and exclusion criteria, and we will refine the wording based on the feedback if more than one reviewer expresses the same concern.

#### 3.3.3 Data extraction and management

Data will be extracted by two reviewers working independently, using an extraction form in Excel (Microsoft Corporation, 2022), based on the coding template in Supporting Information: Appendix 3. The form will be piloted with ten articles on studies with diverse designs and outcomes, to check if more questions or categories are required in the form to capture all relevant information on the population, setting, study design, intervention, data collection and analysis, outcomes, and results. Because of the time‐intensive nature of data extraction, we will attempt to resolve discrepancies by consensus between the two reviewers and by consulting a third reviewer if consensus is not reached.

If there are several articles included that report on the same study, two reviewers will perform the extractions using one form each, to consolidate the data reported in these articles. If there are discrepancies in the data reported in different articles on the same study, we will contact the study authors to ask for clarification. If we cannot reach the authors, we will use the data from the articles that present the most complete datasets and statistical analyses.

For multi‐arm studies, we will only include the intervention and control groups that meet our inclusion criteria. However, we will note the presence of the other groups in the ‘Table of characteristics of included studies’.

Based on our preliminary literature review, we do not expect to find crossover designs for GBI interventions, but if we do, we will use data only from the first stage of the study (i.e., before participants are moved into a different study arm) to avoid carry‐over effects.

#### 3.3.4 Assessment of risk of bias in included studies

We will use the risk of bias tool described by Sharma Waddington and Cairncross (2021), which builds on previously developed tools (Eldridge et al., 2016; Higgins Julian et al., 2016; Hombrados & Waddington, 2012; Jimenez et al., 2018; Sterne et al., 2016; Waddington et al., 2017), and combines scoring criteria for randomized and non‐randomized designs so that the quality of studies using both designs can be compared. Risk of bias will be assessed independently by two reviewers, and ratings of “low risk,” “some concerns” or “high risk” will be assigned for each of eight domains: Confounding, Selection bias, Attrition bias, Motivation bias, Performance bias, Measurement error, Analysis reporting, and Unit of analysis error (Sharma & Cairncross, 2021). We will attempt to resolve discrepancies by consensus, but if it is not reached, the higher risk of bias rating of the two reviewers will be used (i.e., the more cautious rating). To calculate an overall risk of bias score, we will convert the ratings to numerical values (0 = *low risk*, 1 = *some concerns*, 2 = *high risk*) and then sum up the values to get an overall score between 0 (*low risk in every domain*) and 16 (*high risk in every domain*).

#### 3.3.5 Measures of treatment effect

For continuous outcomes, we will calculate standardized mean differences (*d*) to estimate effect sizes with 95% confidence intervals using weighted mean differences in natural (raw) units if standard deviations (SDs) are reported. If SDs are not available, we will calculate them using *p* values, *t* values, or confidence intervals (if reported) using the Campbell Collaboration effect size calculator (https://www.campbellcollaboration.org/escalc/html/EffectSizeCalculator-SMD-main.php).

If some studies report only means and not SDs, *p* values, *t* values, or confidence intervals, we will contact the corresponding authors of those studies to see if these statistics can be provided.

To compare effect sizes where two or more studies report on the same outcome but measure it in different ways, we will calculate Hedges’ *g* values to estimate the effect sizes for each study (Borenstein & Hedges Larry, 2019). Compared to Cohen's *d*, Hedges’ method uses a correction factor in calculating the standardized mean difference to reduce bias due to small sample sizes which can exaggerate the effect size. Hedges’ *g* can be used to estimate effect sizes for studies with independent groups, and (using a slightly different formula) for matched group and for pre‐post single‐group studies (Borenstein & Hedges Larry, 2019).

If dichotomous outcomes are reported, we will calculate odds ratios and 95% confidence intervals to compare effect sizes.

For studies that use an interrupted time series (ITS) design, effect sizes can be estimated in two ways: the difference in the slopes of the pre‐ and post‐intervention regression lines, as well as the change in the level of the outcome after the intervention starts. This change is determined by extrapolating the pre‐intervention regression line to the first post‐intervention time point and calculating the ‘distance’ between the two regression lines at that point (Ramsay et al., 2003). Because these effect size estimates are not based on standardized mean differences, the results of ITS studies will be analyzed separately from other study types.

#### 3.3.6 Unit of analysis issues

To minimize unit‐of‐analysis error, we will analyze results separately for different units of analysis: individual, household, and community. GBI studies typically involve individual‐ and household‐level allocation, with pre‐ and post‐intervention measurements.

Cluster designs pose a challenge to comparing effect sizes across studies because the calculation of standardized mean differences is more difficult, due to the within‐cluster and between‐cluster variability (Hedges, 2007). Incorporating the total variance (within‐ and between‐cluster) and adjusting for baseline measures of outcome variables and covariates can yield a more accurate estimate of effect size (Taylor et al., 2022). We will use a shiny app provided by Taylor and colleagues (https://airshinyapps.shinyapps.io/es_2lvl_clust_adj/) to perform the calculation of effect sizes for cluster‐design studies.

#### 3.3.7 Criteria for determination of independent findings

We expect to find multiple reports of each GBI study; these will be examined as a single study, and we will use all information available. We also expect that some of the multiple reports will be more complete in terms of describing the methodology, and some may report on the primary outcomes we will examine in this review, while others may report on secondary outcomes. If there are differences in the details between reports, we will contact the authors to verify which is correct (e.g., different numbers of participants).

For multiple outcomes in the same study which are conceptually similar, we will choose (in order of preference): (a) the outcome that was classified as a primary outcome for the study, (b) the outcome that was reported first in the abstract, or (c) the outcome that best matches our definition of the construct.

#### 3.3.8 Dealing with missing data

Studies will not be excluded on the basis of how data are reported. If included articles do not report statistical data necessary for meta‐analyses and the data cannot be calculated reliably (e.g., using reported confidence intervals to calculate SDs for continuous outcomes, or using sample sizes and percentages to derive 2 × 2 tables for dichotomous outcomes), we will contact the study authors to ask for the missing data. If we cannot acquire the necessary statistic, the result will not be used in the meta‐analysis, but will be included in the narrative synthesis.

#### 3.3.9 Assessment of heterogeneity

We will use the *I* ^2^ statistic, calculated using RevMan as part of the meta‐analyses, to examine heterogeneity. The *I* ^2^ statistic is a percentage estimate of the variation across studies that is due to heterogeneity and not randomness. An *I* ^2^ value of 0 (zero) would mean there is no inconsistency across the studies, whereas 1 (or 100%) would mean extreme heterogeneity. Studies that markedly increase the *I* ^2^ value will be excluded from meta‐analyses and will be analyzed separately (e.g., if we find that including a fifth study with four others increases *I* ^2^ from 0.30 to 0.75).

#### 3.3.10 Assessment of reporting biases

Because GBI interventions are typically conducted by governments (municipal, state/provincial, federal), we expect that reports will be published both in journals and non‐academic sources, and that this will not be associated with publication bias.

To determine if outcomes are selectively reported or omitted, we will compare different articles on each study, and we will also search for proposals, pre‐analysis plans, and protocols, to see if they specify unreported outcomes.

We will also check for selective outcome and analysis reporting using the risk of bias tool described above.

#### 3.3.11 Data synthesis

##### Quantitative synthesis

Extracted data will be entered into RevMan by one reviewer, and a second reviewer will independently verify the accuracy of the entered data.

Experimental (RCT) and quasi‐experimental studies will be analyzed separately.

If there is sufficient and appropriate data to conduct meta‐analyses (i.e., two or more studies with the same design reporting on the same outcome), we will calculate the pooled effect size using RevMan. The *I* ^2^ statistic will be used to assess heterogeneity between studies. If the *I* ^2^ value is small (≤ 25%), indicating low heterogeneity (Higgins, 2003), we will use a fixed effects model for the meta‐analysis. If *I* ^2^ is larger than 25%, indicating moderate or high heterogeneity, we will use a random effects model. For random effects meta‐analyses, we will estimate the heterogeneity among the effect size parameters using the between‐study variance, *τ* ^2^, which will also be calculated using RevMan.

For multi‐arm studies with a single control group, we will divide the number of participants in the control group by the number of eligible intervention groups, to prevent double counting of participants if more than one intervention group is included in the meta‐analysis.

##### Narrative synthesis

Due to the variation across GBI interventions, study designs, populations, and outcome measures, we expect that meta‐analyses will not be possible for many of the studies. In this case, we will present the findings of these studies in narrative form, including calculated effect sizes for each study. We will construct tables to classify the studies according to the type of GBI, study design, and outcomes. We will also illustrate effect sizes in graphical form for studies that can be grouped and compared in a meaningful way.

#### 3.3.12 Subgroup analysis and investigation of heterogeneity

We will conduct subgroup analyses according to the study design (cluster randomized controlled trials [cRCTs], controlled before and after [CBA], etc.), study duration (<2 years, 2‐4 years, >4 years), generosity of GBI benefits (relative to the official poverty line), individual/household level payment modality, poverty level threshold for eligibility (e.g., income below official poverty line, no income threshold), and take‐back rate if there is additional income from other sources.

To examine whether GBI interventions impact health inequities within the sample populations, we will assess the effects of GBI on physical and mental health across the sociodemographic categories of the PROGRESS‐Plus framework. The PROGRESS acronym stands for place of residence, race/ethnicity, occupation, gender, religion, education, social capital, and socioeconomic status, while “Plus” refers to any other factors which may be associated with disadvantage, such as age, criminal record, disability, or sexual orientation (Kavanagh et al., 2009; O'Neill et al., 2014). Depending on the context of the study, a “Plus” factor may be the most relevant (O'Neill et al., 2014).

If we conduct other post hoc subgroup analyses not specified above or in Supporting Information: Appendix 4 (Table of subgroup and moderator variable codes), we will report in the review that the additional analyses are post hoc and exploratory in nature.

If there are enough included studies to meaningfully compare the difference in effect across subgroups, we will conduct a meta‐regression to test the mean difference between the groups.

As described above, we will use the *I* ^2^ statistic calculated using RevMan to examine heterogeneity. We will investigate the reasons for heterogeneity by exploring and comparing the intervention types and contexts, populations, and outcome measures.

#### 3.3.13 Sensitivity analysis

If there are sufficient similar studies to conduct meta‐analyses, we will verify the robustness of the meta‐analyses by comparing the quality of the studies (as determined by our risk of bias assessments) to ensure that the effect sizes were not excessively influenced by one or more low‐quality studies.

#### 3.3.14 Treatment of qualitative research

We do not plan to include qualitative research.

#### 3.3.15 Summary of findings and assessment of the certainty of the evidence

We will present a GRADE “summary of findings” table and will assess the certainty of the evidence, following the method of Schünemann and colleagues (Schünemann et al., 2019). Separate tables will be presented for each type of GBI intervention (e.g., subsistence‐level benefits for households, monthly amount below €500 for individuals), and the tables will include all the primary and secondary outcomes (listed above) for which results are reported in the included studies.

Two reviewers will independently apply the GRADE approach to assign for each outcome an overall level of the quality of evidence—that is, our level of certainty that the estimate of the effect is close to the true effect. The quality of the evidence will be ranked as “high,” “moderate,” low, or “very low.”

If there is a high degree of heterogeneity among studies so that we cannot pool the results, we will apply the GRADE approach to assess the certainty of evidence and will present a narrative summary of the effect.

## CONTRIBUTIONS OF AUTHORS

- Content: Anita Rizvi, Marcia Gibson, Christina Pollard
- Systematic review methods: Vivian Welch, Elizabeth Kristjansson
- Statistical analysis: George A. Wells
- Information retrieval: Patrick R. Labelle

## DECLARATIONS OF INTEREST

VW is editor‐in‐chief and acting CEO of the Campbell Collaboration. VW will not be involved in the editorial decision process for this review.

MG led a scoping review of public health effects of interventions similar to basic income, published in 2020 in Lancet Public Health.

The authors of this review declare no conflicts of interest.

## PRELIMINARY TIMEFRAME

The approximate date for submission of the systematic review is December 2022.

## PLANS FOR UPDATING THIS REVIEW

We plan to update this review 4 years after publication of the review. If this is not possible for some reason, the lead author will communicate this to the Social Welfare Coordinating Group.

## SOURCES OF SUPPORT

**Internal sources**

• No sources of support provided

**External sources**

• No sources of support provided

## Supporting information

Supplementary information.

## References

[cl21281-bib-0003] Alston, P. (2017). *Universal basic income as a social rights‐based antidote to growing economic insecurity*. Social Science Research Network.

[cl21281-bib-0004] Aubry, T. , Bloch, G. , Brcic, V. , Saad, A. , Magwood, O. , Abdalla, T. , Alkhateeb, Q. , Xie, E. , Mathew, C. , Hannigan, T. , Costello, C. , Thavorn, K. , Stergiopoulos, V. , Tugwell, P. , & Pottie, K. (2020). Effectiveness of permanent supportive housing and income assistance interventions for homeless individuals in high‐income countries: A systematic review. The Lancet Public Health, 5(6), e342–e360. 32504587 10.1016/S2468-2667(20)30055-4

[cl21281-bib-0005] Barder, O. M. (2009). What is poverty reduction? (Working Paper, 170). *Center for Global Development*. https://citeseerx.ist.psu.edu/viewdoc/download?doi=10.1.1.393.7930&rep=rep1&type=pdf

[cl21281-bib-0006] Bidadanure, J. , Kline, S. , Moore, C. , Rainwater, B. , & Thomas, C. (2018). *Basic income in cities: A Guide to city experiments and pilot projects*. National League of Cities. https://www.nlc.org/wp-content/uploads/2020/10/BasicIncomeInCities_Report_For-Release-.pdf

[cl21281-bib-0007] Bidadanure, J. U. (2019). The political theory of universal basic income. Annual Review of Political Science, 22(1), 481–501.

[cl21281-bib-0008] Basic Income Earth Network (BIEN) . (2020). *About basic income*. https://basicincome.org/about-basic-income/

[cl21281-bib-0009] Boozary, A. S. , & Shojania, K. G. (2018). Pathology of poverty: The need for quality improvement efforts to address social determinants of health. BMJ Quality & Safety, 27(6), 421–424. 10.1136/bmjqs-2017-007552 29511090

[cl21281-bib-0010] Borenstein, M. , & Hedges Larry, V. (2019). Effect sizes for meta‐analysis. In H. Cooper , L. V. Hedges , & J. C. Valentine (Eds.), The handbook of research synthesis and meta‐analysis (3rd ed., pp. 208–242). Russell Sage Foundation.

[cl21281-bib-0011] Caputo, R. K. (2007). Working and poor: A panel study of maturing adults in the U.S. Families in Society: The Journal of Contemporary Social Services, 88(3), 351–359.

[cl21281-bib-0012] Carey, M. , & Bell, S. (2020). Universal credit, lone mothers and poverty: Some context and challenges for social work with children and families. Critical and Radical Social Work, 8(2), 189–203.

[cl21281-bib-0013] Centre for Social Justice . (2018). *Universal basic income: An effective policy for poverty reduction?* https://www.centreforsocialjustice.org.uk/wp-content/uploads/2018/08/CSJ_UBI_August-2018.pdf

[cl21281-bib-0014] Cutillo, A. , Raitano, M. , & Siciliani, I. (2020). Income‐based and consumption‐based measurement of absolute poverty: Insights from Italy. Social Indicators Research, 161, 689–710.

[cl21281-bib-0015] Day, C. (2017). *The Beveridge report and the foundations of the welfare state*. The National Archives blog.

[cl21281-bib-0016] Department for Environment, Food & Rural Affairs . (2021). *United Kingdom food security report 2021: Theme 4: Food security at household level*. GOV.UK.

[cl21281-bib-0017] Eldridge, S. , Campbell, M. , Campbell, M. , Drahota‐Towns, A. , Giraudeau, B. , Higgins, J. , Reeves, B. , & Siegfried, N. (2016). *Revised Cochrane risk of bias tool for randomized trials (RoB 2.0): Additional considerations for cluster‐randomized trials*. riskofbias.info.

[cl21281-bib-0018] Economic Policy Institute (EPI) . (2021). The productivity–pay gap. Economic Policy Institute.

[cl21281-bib-0019] Food Banks Canada . (2019). *HungerCount 2019*. https://www.foodbankscanada.ca/getmedia/496dff0c-fe21-46b5-bc71-bd4c20210e6a/HungerCount-2019_FINAL.pdf.aspx?ext=.pdf

[cl21281-bib-0020] Gentilini, U. , Grosh, M. , Rigolini, J. , & Yemtsov, R. (2020). Exploring universal basic income: A guide to navigating concepts, evidence, and practices. World Bank.

[cl21281-bib-0021] Gibson, M. , Hearty, W. , & Craig, P. (2020). The public health effects of interventions similar to basic income: A scoping review. The Lancet Public Health, 5(3), e165–e176. 32113520 10.1016/S2468-2667(20)30005-0 PMC7208547

[cl21281-bib-0022] Gregory Christian, A. , & Coleman‐Jensen, A. (2017). Food insecurity, chronic disease, and health among working‐age adults. United States Department of Agriculture.

[cl21281-bib-0023] Guio, A. C. , Marlier, E. , Gordon, D. , Fahmy, E. , Nandy, S. , & Pomati, M. (2016). Improving the measurement of material deprivation at the European Union level. Journal of European Social Policy, 26(3), 219–333.

[cl21281-bib-0024] Gundersen, C. , & Ziliak, J. P. (2015). Food insecurity and health outcomes. Health Affairs, 34(11), 1830–1839. 26526240 10.1377/hlthaff.2015.0645

[cl21281-bib-0025] Gupta, R. , Jacob, J. , & Bansal, G. (2021). The role of UBI in mitigating the effects of psychosocial stressors: A review and proposal. Psychological Reports, 125, 1801–1823. 33789535 10.1177/00332941211005115

[cl21281-bib-0026] Gupta, B. S. , & Theoharis, L. (2020). *US poverty measure is misleading, leaves out millions of Americans who need help*. Business Insider.

[cl21281-bib-0027] Günther, S. (2020). Basic income: A systematic review of existing programs and their effects. Umeå School of Business and Economics, Umeå University.

[cl21281-bib-0028] Hasdell, R. (2020). What we know about Universal Basic Income: A cross‐synthesis of reviews. Basic Income Lab.

[cl21281-bib-0029] Hedges, L. V. (2007). Effect sizes in cluster‐randomized designs. Journal of Educational and Behavioral Statistics, 32(4), 341–370.

[cl21281-bib-0030] Higgins, J. P. T. (2003). Measuring inconsistency in meta‐analyses. BMJ, 327(7414), 557–560. 12958120 10.1136/bmj.327.7414.557 PMC192859

[cl21281-bib-0031] Higgins, J. P. T. , Sterne Jonathan, A. C. , Savovic, J. , Page Matthew, J. , Hróbjartsson, A. , Boutron, I. , Reeves, B. , & Eldridge, S. (2016). A revised tool for assessing risk of bias in randomized trials. Cochrane Database of Systematic Reviews, 10(Suppl. 1), 29–31.

[cl21281-bib-0032] Hombrados, J. G. , & Waddington, H. (2012). *A tool to assess risk of bias for experiments and quasi‐experiments in development research*. International Initiative for Impact Evaluation (3ie).

[cl21281-bib-0033] Hoynes, H. , & Rothstein, J. (2019). Universal basic income in the United States and advanced countries. Annual Review of Economics, 11(1), 929–958.

[cl21281-bib-0034] Hunger + Health . (2022). *What is food insecurity in America?*

[cl21281-bib-0035] International Labour Organization . (2016). *World employment and social outlook 2016—Transforming jobs to end poverty* (Vol. 192). http://www.ilo.org/wcmsp5/groups/public/@dgreports/@dcomm/@publ/documents/publication/wcms_481534.pdf

[cl21281-bib-0036] International Monetary Fund . (2022). Statistical appendix. In World economic outlook: War sets back the global recovery (pp. 101–127). International Monetary Fund. https://www.imf.org/-/media/Files/Publications/WEO/2021/April/English/stasapp.ashx

[cl21281-bib-0037] Interagency Technical Working Group (ITWG) . (2021). *Final report of the Interagency Technical Working Group on Evaluating Alternative Measures of Poverty*. U.S. Bureau of Labor Statistics. https://www.bls.gov/evaluation/final-report-of-the-interagency-technical-working-group-on-evaluating-alternative-measures-of-poverty.pdf

[cl21281-bib-0038] Jenkins, B. (2019). A guaranteed basic income and the aesthetics of existence. Journal of Cultural Economy, 12(1), 21–35.

[cl21281-bib-0039] Jimenez, E. , Waddington, H. , Goel, N. , Prost, A. , Pullin, A. , White, H. , Lahiri, S. , & Narain, A. (2018). Mixing and matching: Using qualitative methods to improve quantitative impact evaluations (IEs) and systematic reviews (SRs) of development outcomes. Journal of Development Effectiveness, 10(4), 400–421.

[cl21281-bib-0040] Kavanagh, J. , Oliver, S. , Caird, J. , Tucker, H. , Greaves, A. , Oakley, A. , Harden, A. , Lorenc, T. , & Thomas, J. (2009). Inequalities and the mental health of young people: A systematic review of secondary school‐based cognitive behavioural interventions. EPPI‐Centre, Social Science Research Unit, Institute of Education, University of London.

[cl21281-bib-0041] Kenworthy, L. (1999). Do social‐welfare policies reduce poverty? A cross‐national assessment. Social Forces, 77(3), 1119–1139.

[cl21281-bib-0042] Koebel, K. , & Pohler, D. (2019). Expanding the Canada workers benefit to design a guaranteed basic income. Canadian Public Policy, 45(3), 283–309.

[cl21281-bib-0043] Konle‐Seidl, R. A. (2021). Strengthening minimum income protection in the EU. Publications Office of the EU.

[cl21281-bib-0044] Laín, B. (2019). *Report on the preliminary results of the B‐MINCOME project (2017‐2018): Combining a guaranteed minimum income and active social policies in deprived urban areas of Barcelona*. Planning and Innovation Department Area of Social Rights, Barcelona City Council.

[cl21281-bib-0045] Loopstra, R. (2018). Interventions to address household food insecurity in high‐income countries. Proceedings of the Nutrition Society, 77(3), 270–281. 29580316 10.1017/S002966511800006X

[cl21281-bib-0046] Loopstra, R. , & Tarasuk, V. (2013). What does increasing severity of food insecurity indicate for food insecure families? Relationships between severity of food insecurity and indicators of material hardship and constrained food purchasing. Journal of Hunger & Environmental Nutrition, 8(3), 337–349.

[cl21281-bib-0047] McDowell, T. , & Ferdosi, M. (2021). The impacts of the Ontario basic income pilot: A comparative analysis of the findings from the hamilton region. Basic Income Studies, 16(2), 209–256.

[cl21281-bib-0048] McGowan, J. , Sampson, M. , Salzwedel, D. M. , Cogo, E. , Foerster, V. , & Lefebvre, C. (2016). PRESS peer review of electronic search strategies: 2015 guideline statement. Journal of Clinical Epidemiology, 75, 40–46. 10.1016/j.jclinepi.2016.01.021 27005575

[cl21281-bib-0049] McLeod, L. , & Veall, M. (2006). The dynamics of food insecurity and overall health: Evidence from the Canadian National Population Health Survey. Applied Economics, 38(18), 2131–2146.

[cl21281-bib-0050] Merrill, R. , Neves, C. , & Laín, B. (2022). How the findings help advance the basic income debate and advocacy. In R. Merrill , C. Neves , & B. Laín (Eds.), Basic income experiments: A critical examination of their goals, contexts, and methods (pp. 173–205). Springer International Publishing.

[cl21281-bib-0051] The Methods Group of the Campbell Collaboration . (2019). *Methodological expectations of Campbell Collaboration intervention reviews: Conduct standards*. Campbell Policies and Guidelines.

[cl21281-bib-0052] The Methods Group of the Campbell Collaboration . (2019). *Methodological expectations of Campbell Collaboration intervention reviews: Reporting standards*. Campbell Policies and Guidelines.

[cl21281-bib-0053] Meyer, B. D. , & Sullivan, J. X. (2012). Identifying the disadvantaged: Official poverty, consumption poverty, and the new supplemental poverty measure. Journal of Economic Perspectives, 26(3), 111–136.

[cl21281-bib-0054] Microsoft Corporation . (2022). *Microsoft Excel Spreadsheet Software—Microsoft 365*. Microsoft.

[cl21281-bib-0055] Nettle, D. , Johnson, E. , Johnson, M. , & Saxe, R. (2021). Why has the COVID‐19 pandemic increased support for Universal Basic Income? Humanities and Social Sciences Communications, 8(1), 79.

[cl21281-bib-0056] Nilsson, S. F. , Nordentoft, M. , & Hjorthøj, C. (2019). Individual‐level predictors for becoming homeless and exiting homelessness: A systematic review and meta‐analysis. Journal of Urban Health, 96(5), 741–750. 31388823 10.1007/s11524-019-00377-x PMC6814700

[cl21281-bib-0057] O'Neill, J. , Tabish, H. , Welch, V. , Petticrew, M. , Pottie, K. , Clarke, M. , Evans, T. , Pardo Pardo, J. , Waters, E. , White, H. , & Tugwell, P. (2014). Applying an equity lens to interventions: Using PROGRESS ensures consideration of socially stratifying factors to illuminate inequities in health. Journal of Clinical Epidemiology, 67, 56–64. 24189091 10.1016/j.jclinepi.2013.08.005

[cl21281-bib-0058] Organisation for Economic Co‐operation and Development . (2019). A declining middle‐income class? In *Under pressure: The squeezed middle class*.

[cl21281-bib-0059] Organisation for Economic Co‐operation and Development . (2020). Social expenditure database (SOCX). OECD—Better Policies for Better Lives.

[cl21281-bib-0060] Organisation for Economic Co‐operation and Development . (2021a). Perspectives on global development 2021: From protest to progress?

[cl21281-bib-0061] OECD . (2021b). *Poverty rate (indicator)*. http://data.oecd.org/inequality/poverty-rate.htm

[cl21281-bib-0062] Organisation for Economic Co‐operation and Development . (2022). Poverty rate (indicator).

[cl21281-bib-0063] Orrell, B. (2021). Let them spend cash? American Enterprise Institute (AEI).

[cl21281-bib-0064] Pattaro, S. , Bailey, N. , Williams, E. , Gibson, M. , Wells, V. , Tranmer, M. , Dibben, C. (2022). The impacts of benefit sanctions: A scoping review of the quantitative research evidence. Journal of Social Policy, 51(3), 611–653. 36000019 10.1017/S0047279421001069 PMC7613403

[cl21281-bib-0065] Peer, A. (2021). Global poverty: Facts, FAQs, and how to help. World Vision.

[cl21281-bib-0066] Pinto, A. D. , Perri, M. , Pedersen, C. L. , Aratangy, T. , Hapsari, A. P. , Hwang, S. W. (2021). Exploring different methods to evaluate the impact of basic income interventions: A systematic review. International Journal for Equity in Health, 20(142), 142. 10.1186/s12939-021-01479-2 34134715 PMC8206888

[cl21281-bib-0067] Policy Options . (2004). *New century, new risks: The Marsh Report and the post‐war welfare state in Canada*. https://policyoptions.irpp.org/magazines/social-policy-in-the-21st-century/new-century-new-risks-the-marsh-report-and-the-post-war-welfare-state-in-canada/

[cl21281-bib-0068] Poverty Analysis Discussion Group . (2012). Understanding poverty and wellbeing: A note with implications for research and policy. Department for International Development (DFID).

[cl21281-bib-0069] Power, E. , Abercrombie, D. , Fafard St‐Germain, A.‐A. , & Vanderkooy, P. (2016). *Prevalence, severity and impact of household food insecurity: A serious public health issue*. Dietitians of Canada. https://www.dietitians.ca/DietitiansOfCanada/media/Documents/Resources/HFI-Background-DC-FINAL.pdf?ext=.pdf

[cl21281-bib-0070] Ramsay, C. R. , Matowe, L. , Grilli, R. , Grimshaw, J. M. , & Thomas, R. E. (2003). Interrupted time series designs in health technology assessment: Lessons from two systematic reviews of behavior change strategies. International Journal of Technology Assessment in Health Care, 19(4), 613–623. 15095767 10.1017/s0266462303000576

[cl21281-bib-0071] Ramsey, R. , Giskes, K. , Turrell, G. , & Gallegos, D. (2011). Food insecurity among Australian children: Potential determinants, health and developmental consequences. Journal of Child Health Care, 15(4), 401–416. 22199175 10.1177/1367493511423854

[cl21281-bib-0072] Reed, H. , & Lansley, S. (2016). *Universal Basic Income: An idea whose time has come?* Compass.

[cl21281-bib-0073] Riches, G. , & Tarasuk, V. (2014). Canada: Thirty years of food charity and public policy neglect. In G. Riches , & T. Silvasti (Eds.), First world hunger revisited: Food charity or the right to food? (pp. 42–56). Palgrave Macmillan UK.

[cl21281-bib-0074] Robson, J. , & Schwartz, S. (2020). Who doesn't file a tax return? A portrait of non‐filers. Canadian Public Policy, 46(3), 323–339.

[cl21281-bib-0075] Romano, S. (2015). Idle paupers, scroungers and shirkers: Past and new social stereotypes of the undeserving welfare claimant in the UK. In L. Foster , A. Brunton , C. Deeming , & T. Haux (Eds.), Defence of welfare II (pp. 65–68). Policy Press.

[cl21281-bib-0076] Rose, J. (1985). From Bismark to Roosevelt: How the welfare began. Scholastic Update, 118, 13–16.

[cl21281-bib-0077] Sarabia, D. N. (2016). Consumption: The best way to measure poverty? *BORGEN Magazine*.

[cl21281-bib-0078] Schenck‐Fontaine, A. , & Panico, L. (2019). Many kinds of poverty: Three dimensions of economic hardship, their combinations, and children's behavior problems. Demography, 56(6), 2279–2305. 31808103 10.1007/s13524-019-00833-y PMC7172985

[cl21281-bib-0079] Schünemann, H. J. , Higgins, J. P. T. , Vist, G. E. , Glasziou, P. , Akl, E. A. , Skoetz, N. , Guyatt, G. H. , the Cochrane GRADEing Methods Group (formerly Applicability and Recommendations Methods Group), & the Cochrane Statistical Methods Group . (2019). Completing ‘Summary of findings’ tables and grading the certainty of the evidence. In J. P. T. Higgins , J. Thomas , J. Chandler , M. Cumpston , T. Li , M. J. Page , & V. A. Welch , (Eds.), Cochrane handbook for systematic reviews of interventions (pp. 375–402). John Wiley & Sons, Ltd.

[cl21281-bib-0080] Sustainable Development Solutions Network (SDSN) . (2019). *Multidimensional poverty index*.

[cl21281-bib-0081] Seligman, H. K. , & Schillinger, D. (2010). Hunger and socioeconomic disparities in chronic disease. New England Journal of Medicine, 363(1), 6–9. 20592297 10.1056/NEJMp1000072

[cl21281-bib-0082] Sharma, W. H. , & Cairncross, S. (2021). PROTOCOL: water, sanitation and hygiene for reducing childhood mortality in low‐ and middle‐income countries. Campbell Systematic Reviews, 17(1), e1135. 10.1002/cl2.1135 PMC8356349 37050969

[cl21281-bib-0083] Shea, B. J. , Reeves, B. C. , Wells, G. , Thuku, M. , Hamel, C. , Moran, J. , Moher, D. , Tugwell, P. , Welch, V. , Kristjansson, E. , & Henry, D. A. (2017). AMSTAR 2: A critical appraisal tool for systematic reviews that include randomised or non‐randomised studies of healthcare interventions, or both. BMJ, 358, j4008. 28935701 10.1136/bmj.j4008 PMC5833365

[cl21281-bib-0084] Standing, G. (2019). Basic income as common dividends: Piloting a transformative policy. Progressive Economy Forum.

[cl21281-bib-0085] Standing, G. (2021). Basic income pilots: Uses, limitations and design principles. Basic Income Studies, 16(1), 75–99.

[cl21281-bib-0086] Statistics Canada . (2021). Dimensions of poverty hub.

[cl21281-bib-0087] Sterne, J. A. , Hernán, M. A. , Reeves, B. C. , Savović, J. , Berkman, N. D. , Viswanathan, M. , Henry, D. , Altman, D. G. , Ansari, M. T. , Boutron, I. , Carpenter, J. R. , Chan, A. W. , Churchill, R. , Deeks, J. J. , Hróbjartsson, A. , Kirkham, J. , Jüni, P. , Loke, Y. K. , Pigott, T. D. , … Higgins, J. P. (2016). ROBINS‐I: A tool for assessing risk of bias in non‐randomised studies of interventions. BMJ, 355, i4919. 27733354 10.1136/bmj.i4919 PMC5062054

[cl21281-bib-0088] Stone, C. , Trisi, D. , Sherman, A. , & Beltrán, J. (2020). A guide to statistics on historical trends in income inequality. Center on Budget and Policy Priorities.

[cl21281-bib-0089] Tarasuk, V. , Fafard St‐Germain, A.‐A. , & Loopstra, R. (2020). The relationship between food banks and food insecurity: Insights from Canada. VOLUNTAS: International Journal of Voluntary and Nonprofit Organizations, 31(5), 841–852. 10.1007/s11266-019-00092-w

[cl21281-bib-0090] Tarasuk, V. , & Mitchell, A. (2020). *Household food insecurity in Canada, 2017‐18. Research to identify policy options to reduce food insecurity (PROOF)*. https://proof.utoronto.ca/wp-content/uploads/2020/03/Household-Food-Insecurity-in-Canada-2017-2018-Full-Reportpdf.pdf]

[cl21281-bib-0091] Taylor, J. A. , Pigott, T. , & Williams, R. (2022). Promoting knowledge accumulation about intervention effects: Exploring strategies for standardizing statistical approaches and effect size reporting. Educational Researcher, 51(1), 72–80.

[cl21281-bib-0092] Thoits, P. A. (2010). Stress and health: Major findings and policy implications. Journal of Health and Social Behavior, 51(1_suppl), S41–S53. 20943582 10.1177/0022146510383499

[cl21281-bib-0093] Thomas, M. M. C. , Miller, D. P. , & Morrissey, T. W. (2019). Food insecurity and child health. Pediatrics, 144(4), e20190397. 31501236 10.1542/peds.2019-0397

[cl21281-bib-0094] Toppenberg, L. (2017). *Exploring food insecurity policy through multidimensional poverty measures*. Texas Medical Center Dissertations (via ProQuest), pp. 1–71.

[cl21281-bib-0095] Trattner, W. I. (2007). From poor law to welfare state: A history of social welfare in America. Simon and Schuster.

[cl21281-bib-0096] United Kingdom Government . (2014). *Universal credit*. GOV.UK.

[cl21281-bib-0097] United Kingdom Government . (2022). *How to claim tax credits*. GOV.UK.

[cl21281-bib-0098] United Nations Department of Economic and Social Affairs (UN DESA) . (2022). *Statistical Annex—Country classifications*. World Economic Situation and Prospects. United Nations, 2022:163‐171.

[cl21281-bib-0099] United Nations . (2020). *Inequality—Bridging the divide*. UN75: 2020 and beyond 2020.

[cl21281-bib-0100] Van Mechelen, N. , & Janssens, J. (2017). *Who is to blame? An overview of the factors contributing to the non‐take‐up of social rights*. Herman Deleeck Centre for Social Policy, University of Antwerp. https://repository.uantwerpen.be/docman/irua/cf28ca/147576.pdf

[cl21281-bib-0101] Van Parijs, P. (2004). Basic income: A simple and powerful idea for the twenty‐first century. Politics & Society, 32(1), 7–39.

[cl21281-bib-0102] Van Parijs, P. , & Vanderborght, Y. (2017). Basic income: A radical proposal for a free society and a sane economy. Harvard University Press.

[cl21281-bib-0103] Versace, C. , Hawkins, L. E. , & Abssy, M. (2021). World reimagined: The rise of the global middle class. Nasdaq.

[cl21281-bib-0104] Waddington, H. , Aloe, A. M. , Becker, B. J. , Djimeu, E. W. , Hombrados, J. G. , Tugwell, P. , Wells, G. , & Reeves, B. (2017). Quasi‐experimental study designs series—Paper 6: Risk of bias assessment. Journal of Clinical Epidemiology, 89, 43–52. 28351693 10.1016/j.jclinepi.2017.02.015

[cl21281-bib-0105] Welch, V. , Petticrew, M. , Tugwell, P. , Moher, D. , O'Neill, J. , Waters, E. , White, H. , & PRISMA‐Equity Bellagio Group . (2012). PRISMA‐equity 2012 extension: Reporting guidelines for systematic reviews with a focus on health equity. PLoS Medicine, 9(10), e1001333. 23222917 10.1371/journal.pmed.1001333 PMC3484052

[cl21281-bib-0106] Wheeler, B. (2015). How did we get to this welfare state? *BBC News*.

[cl21281-bib-0107] Whelan, C. T. , Nolan, B. , & Maître, B. (2014). Multidimensional poverty measurement in Europe: An application of the adjusted headcount approach. Journal of European Social Policy, 24(2), 183–197.

[cl21281-bib-0108] Widerquist, K. , Noguera, J. , Vandeborght, Y. , & Wispelaere, J. (Eds.). (2013). Basic income: An Anthology of Contemporary Research. Wiley‐Blackwell.

[cl21281-bib-0109] Winchester, N. (2021). *Universal credit: an end to the uplift*. UK Parliament—House of Lords Library.

[cl21281-bib-0110] Wolfson, M. (2018). *How a guaranteed income could work*. Policy Options.

[cl21281-bib-0111] World Bank Group . (2022). World Bank country and lending groups—World Bank data help desk. World Bank Group. https://datahelpdesk.worldbank.org/knowledgebase/articles/906519-world-bank-country-and-lending-groups

[cl21281-bib-0112] Yang, J. , Mohan, G. , Pipil, S. , & Fukushi, K. (2021). Review on basic income (BI): Its theories and empirical cases. Journal of Social and Economic Development, 23, 203–239.

