# Supplementary material for: PROTOCOL: Effects of guaranteed basic income interventions on poverty‐related outcomes in high‐income countries: A systematic review
Source: Campbell Syst Rev. 2022 Oct 6;18(4):e1281. doi: 10.1002/cl2.1281 (PMC9538708; doi:10.1002/cl2.1281)
Supplement: Supplementary file 1 — Supplementary information. [file CL2-18-e1281-s001.docx]

# Appendices

## 1 MEDLINE(Ovid) search strategy

MEDLINE (Ovid; 1946 to May 13, 2022)

| **#** | **Searches** | **Results** |
| --- | --- | --- |
| 1 | ("basic income").ti,ab,kf. | 73 |
| 2 | ((guarantee* or universal*) adj2 (income or allowance*)).ti,ab,kf. | 135 |
| 3 | (minim* adj2 income).ti,ab,kf. | 171 |
| 4 | (citizen* adj2 (income or dividend*)).ti,ab,kf. | 82 |
| 5 | ("negative income tax*").ti,ab,kf. | 4 |
| 6 | (optimal adj2 income).ti,ab,kf. | 13 |
| 7 | (income adj2 maintenance).ti,ab,kf. | 79 |
| 8 | (unconditional adj2 (cash or transfer*)).ti,ab,kf. | 107 |
| 9 | mincome*.ti,ab,kf. | 4 |
| 10 | demogrant*.ti,ab,kf. | 2 |
| 11 | or/1-10 | 610 |

## 2 Table of study inclusion and exclusion criteria

|  | **Include** | **Exclude** |
| --- | --- | --- |
| **Article type** | Primary research | - any literature that synthesizes, summarizes or refers to the results of primary research - e.g. reviews, compilations, news and magazine articles, editorials, opinion pieces, textbook chapters, blogs  - proposals for studies, programs or policies |
| **Intervention** | Any cash transfer intervention for adults (18+ years old) that: (1) is unconditional, (2) has regular payment intervals, (3) provides a guaranteed minimum amount, and (4) provides fixed or predictable amounts | - interventions that are conditional - e.g. require having or seeking employment, taking training courses, participating in educational or counselling programs, having children of a certain age, caring for adults, having a disability  - unconditional cash transfers of amounts that vary by more than 10% during the study period due to changes in program funding/budget |
| **Study design** | Any design that compares quantitative pre- and post-intervention data  Multi-arm studies if one or more intervention arms meet the inclusion criteria | - cross-sectional studies using data from a single time point  - interrupted time series (ITS) with less than three time points before and three time points after the intervention  - qualitative studies - e.g. case reports, interviews, focus groups |
| **Setting** | Any setting in high-income countries classified as developed by UN DESA* | Not in a developed high-income country* |
| **Participants** | Anyone in a developed high-income country (including children of GBI recipients) | No restrictions |
|  |  |  |

*Developed high-income countries are listed in Table A of the Statistical Annex of *World Economic Situation and Prospects 2022* (https://www.un.org/development/desa/dpad/wp-content/uploads/sites/45/WESP2022_ANNEX.pdf)

## 3 Data extraction template

| **General information** | |
| --- | --- |
| Covidence ID# |  |
| Reviewer's initials |  |
| First author's last name and year |  |
| Corresponding author's contact info |  |
| Source (e.g. journal name, organization/government name) |  |
| Publication type | Select from: journal article, report, other (please specify) |
| Country | Select from: Canada, Finland, France, Germany, Italy, Japan, Netherlands, United Kingdom, Spain, United States, other (please specify) |
| Region (city, state/province, or region described in study) |  |
| Study start date (MM/YYYY) |  |
| Study end date (MM/YYYY) |  |
| Duration of follow-up (baseline to final measurement) |  |
| Study objectives (if presented) |  |
| Main conclusions (as reported) |  |
| Funder |  |
| Ethics approval |  |
| **Methods** | |
| Study design | Select from: RCT, cRCT, before-after (w/o control), CBA, RDD, ITS, other (please specify) |
| Statistical methods used | (Can use article text with page numbers) |
| Number of intervention arms |  |
| Description of intervention(s) | (Can use article text with page numbers) |
| Type of control/comparison | Select from: no intervention, other intervention, no control/comparison group, other control (please specify) |
| Allocation type | Select from: individual, household |
| Units of analysis | Individual, household and/or community level |
| Sociodemographic characteristics used to describe sample and/or compare groups at baseline |  |
| Sociodemographic characteristics used in subgroup analyses of intervention effects |  |
| **Population** | |
| Setting (e.g. urban, low-income neighbourhood, rural) |  |
| Age (mean, SD, range, as reported) |  |
| Sociodemographic characteristics for eligibility in study | e.g. income below x, age between x and y, sex, released from prison |
| Reasons for exclusion from study (if reported) |  |
| Total number of participants at baseline |  |
| Number of participants in each group at baseline |  |
| Number of participants in each group at end of study |  |
| Number of participants lost in each group |  |
| **Outcomes** |  |
| Outcomes reported | e.g. food insecurity, subjective financial stress, education level, self-reported health, material deprivation index score |
| Method of assessment for each outcome | Type of scale, instrument, source of income data or health data, etc. |
| Validity of each measure (if reported) |  |
| Timing of outcome assessment (e.g. baseline, 18 months, 36 months) |  |
| **Results** |  |
| Effect estimates reported for each outcome  (Summarize separately for each intervention if multi-arm study)  (Note if reported effect estimates are adjusted for covariates) | e.g. mean difference and SD, *p*-value or confidence interval |
| Type of effect estimate for each outcome | e.g. standardized mean difference, difference in differences, mean ratio, odds ratio (for dichotomous measures) |
| Statistically significant differences across subgroups for each outcome (and each intervention if multi-arm study) |  |

## 4 Table of subgroup and moderator variable codes

| **Subgroup/variable** | **Types/levels/ranges** |
| --- | --- |
| Study design | RCT, cRCT, quasi-experimental with comparison/control group, pre-post study w/o comparison or control group |
| Study duration | <2 years, 2-4 years, >4 years |
| Allocation level | Individual, household |
| Generosity of GBI benefits  (calculated based on official poverty line (OPL) at time of study; for NIT interventions, based on guaranteed minimum amount and reported strata of amounts received) | Less than half of OPL, 50% to 75% of OPL, 76% to 100% of OPL, higher than OPL |
| Income threshold for intervention eligibility | Below 75% of OPL, 75% to 100% of OPL, above OPL, no income threshold |
| Take-back rate for additional income from other sources | Less than 25%, 25% to 50%, higher than 50% |
| Setting (as reported) | Urban, urban low-income, peri-urban (e.g. suburban, small town), peri-urban low-income, rural, rural low-income |
| Age (including children of intervention recipients) | Under 12, 12 to 17, 18 to 24, 25 to 39, 40 to 65, over 65 |
| Race/ethnicity | (We will compile categories based on the information reported in included studies) |
| Occupation | Routine/low-skilled work, non-routine work, unemployed |
| Sex | Male, female |
| Gender identity | (We will compile categories based on the information reported in included studies) |
| Religion | Christian, Muslim, Jewish, Hindu, Sikh, non-denominational, atheist, other |
| Education | Less than high school, high school completed, some college or university, college or undergraduate degree, graduate degree |
| Ability | Wheelchair used, wheelchair not used, not applicable |
| Social capital | (We will compile categories based on the information reported in included studies) |
| Released from prison | Less than 2 years before study, 2 years or more before study, not applicable |
| Criminal record | Yes, no |

[Enter text here]
